# Supplementary material for: Circulating microRNAs as Biomarkers of Hepatic Fibrosis in Schistosomiasis Japonica Patients in the Philippines
Source: Diagnostics (Basel). 2022 Aug 5;12(8):1902. doi: 10.3390/diagnostics12081902 (PMC9406767; doi:10.3390/diagnostics12081902)
Supplement: Supplementary file 1 [file diagnostics-12-01902-s001.zip › diagnostics-1831667-supplementary.pdf]

Supplementary File S1: Ranges adjusted for body weight in Chinese population.

Body height (cm)	Liver: length of left lobe (mm)			Liver: thickness of left lobe (mm)		
	N	Mean	S.D.	*N*	Mean	S.D.
80–100	70	57.7	7.6	70	43.9	5.5
101–120	43	63.1	8.0	43	42.6	5.4
121–140	71	62.1	8.3	71	46.8	7.0
141–160	175	63.4	10.6	174	50.5	9.5
>160	191	66.6	11.1	191	53.2	9.3
Total	550	63.6	10.4	549	49.5	9.2
	Liver: length of right lobe[a] (mm)			Main portal vein[b] (mm)		
80–100	70	81.6	6.9	70	6.4	0.6.
101–120	42	90.8	6.3	43	6.7	0.5
121–140	71	105.3	7.8	69	7.6	1.0
141–160	173	111.6	9.5	169	8.6	1.4
>160	190	116.1	9.1	188	9.5	1.3
Total	546	106.9	14.6	539	8.3	1.6
	Spleen length (mm)			Spleen thickness (mm)		
80–100	70	61.9	8.2	70	19.2	2.6
101–120	43	67.8	10.3	43	23.3	6.6
121–140	71	80.4	10.7	71	26.3	4.3
141–160	174	86.1	14.7	175	28.4	4.7
>160	190	89.7	16.5	190	29.1	5.9
Total	548	82.1	17.0	549	26.8	6.0
	Portal branch wall thickness[c] (mm)					
80–100	66	1.3	0.4			
101–120	34	1.9	0.3			
121–140	71	2.0	0.4			
141–160	174	2.2	0.6			
>160	190	2.1	0.9			
Total	535	2.0	0.7			

[a] Max oblique diameter.
[b] Inner diameter.
[c] Portal branch wall thickness expressed as external diameter minus diameter of lumen.

Supplementary Tables

Supplementary Table S1. Selected miRNAs based on literature review and initial miRNA profiling.

miRNA	Accession #	Sequences (mature)
Pro-fibrotic miRNAs		
hsa-miR-200b-3p	MIMAT0000318	UAAUACUGCCUGGUAAUGAUGA
hsa-miR-93-5p	MIMAT0000093	CAAAGUGCUGUUCGUGCAGGUAG
Anti-fibrotic miRNAs		
hsa-miR-122-5p	MIMAT0000421	UGGAGUGUGACAAUGGUGUUUG
hsa-miR-146a-5p	MIMAT0000449	UGAGAACUGAAUUCCAUGGGUU
hsa-miR-150-5p	MIMAT0000451	UCUCCCAACCCUUGUACCAGUG
hsa-let-7a-5p	MIMAT0000062	UGAGGUAGUAGGUUGUAUAGUU

Supplementary Table S2. Dynamics of US-detectable hepatic fibrosis severity at baseline and at 6 months after PZQ treatment among the 136 patients enrolled in the cohort.

**Hepatic fibrosis, baseline**[1]	**Hepatic fibrosis, after 6 months**[1]			**Total**	**Regression of Fibrosis Grade (RF)**		**Progression of Fibrosis Grade (PF)**		**Stable Fibrosis Grade (SF)**	
	T0	T1/2	T3		no.	%	no.	(%)	no.	(%)
T0	58	0	0	58	-	-	0	0	58	100.0
T1/2	1	30	0	31	1	3.2	0	0	30	96.7
T3	0	0	47	47	0 (0)	-	-	-	47	100.0
**Total**	59	30	47	136	20	14.7	17	12.5	99	72.8

Note: [1] classification of hepatic fibrosis based on Ohmae et al. (1992); **Abbreviations**: US-detectable hepatic fibrosis types 0 (T0), 1/2 (T1/2), and 3 (T3)

Supplementary Table S3. Dynamics of US-detectable hepatic fibrosis severity at baseline and at 12 months after PZQ treatment among the 136 patients enrolled in the cohort.

**Hepatic fibrosis, baseline[1]**	**Hepatic fibrosis, after 12 months[1]**			**Total**	**Regression of Fibrosis Grade (RF)**		**Progression of Fibrosis Grade (PF)**		**Stable Fibrosis Grade (SF)**	
	T0	T1/2	T3		no.	%	no.	(%)	no.	(%)
T0	52	6	0	58	-	-	6	10.3	52	89.7
T1/2	10	19	2	31	10	32.3	2	6.4	19	61.3
T3	0	0	47	47	0 (0)	-	-	-	47	100.0
**Total**	62	25	49	136	20	14.7	17	12.5	99	72.8

Note: [1] classification of hepatic fibrosis based on Ohmae et al. (1992); **Abbreviations**: US-detectable hepatic fibrosis types 0 (T0), 1/2 (T1/2), and 3 (T3)

Supplementary Table S4. Dynamics of US-detectable hepatic fibrosis severity at baseline and at 24 months after PZQ treatment among the 136 patients enrolled in the cohort.

**Hepatic fibrosis, baseline**[1]	**Hepatic fibrosis, after 24 months**[1]			**Total**	**Regression of Fibrosis Grade (RF)**		**Progression of Fibrosis Grade (PF)**		**Stable Fibrosis Grade (SF)**	
	T0	T1/2	T3		no.	%	no.	(%)	no.	(%)
T0	46	12	0	58	-	-	12	20.7	46	79.3
T1/2	20	6	5	31	20	64.5	5	16.1	6	19.4
T3	0	0	47	47	0 (0)	-	-	-	47	100.0
**Total**	66	18	52	136	20	14.7	17	12.5	99	72.8

Note: [1] classification of hepatic fibrosis based on Ohmae et al. (1992); **Abbreviations**: US-detectable hepatic fibrosis types 0 (T0), 1/2 (T1/2), and 3 (T3)

Supplementary Table S5. Fold change of the 19 differentially expressed miRNAs in the pooled sera of chronic schistosomiasis patients with different degrees of hepatic fibrosis (Sj+|T1–3, *n* = 8) and non-infected participants without hepatic fibrosis (Sj–/T0, n = 8) using chronic schistosomiasis patients without hepatic fibrosis (Sj+/T0, n = 8) as comparator.

miRNA	Fold Change (FC) [1,2,3]	
	Sj+/T1–3	Sj–/T0
**miR-93-5p**	21.56	15.03
miR-27b-3p	15.67	ND
miR-24-3p	12.04	2.30
miR-27a-3p	10.34	0.25
miR-30b-5p	9.45	ND
miR-16-5p	6.77	0.03
miR-30a-5p	6.50	0.37
miR-25-3p	5.82	0.00
miR-30e-5p	5.24	0.19
miR-425-5p	4.59	0.01
let-7g-5p	4.53	0.18
let-7i-5p	4.44	0.01
miR-21-5p	4.32	0.19
**miR-200b-3p**	4.00	2.07
**miR-150-5p**	0.35	0.02
miR-151a-5p	0.33	0.01
**let-7a-5p**	0.22	0.00
**miR-146a-5p**	0.22	0.43
**miR-122-5p**	0.18	0.21

Note: [1] chronic schistosomiasis patients without hepatic fibrosis were used as comparator, [2] results are shown for miRNAs that had significant FC ≥2 or ≤0.5, [3] miRNAs in bold face were individually validated

Supplementary Figures

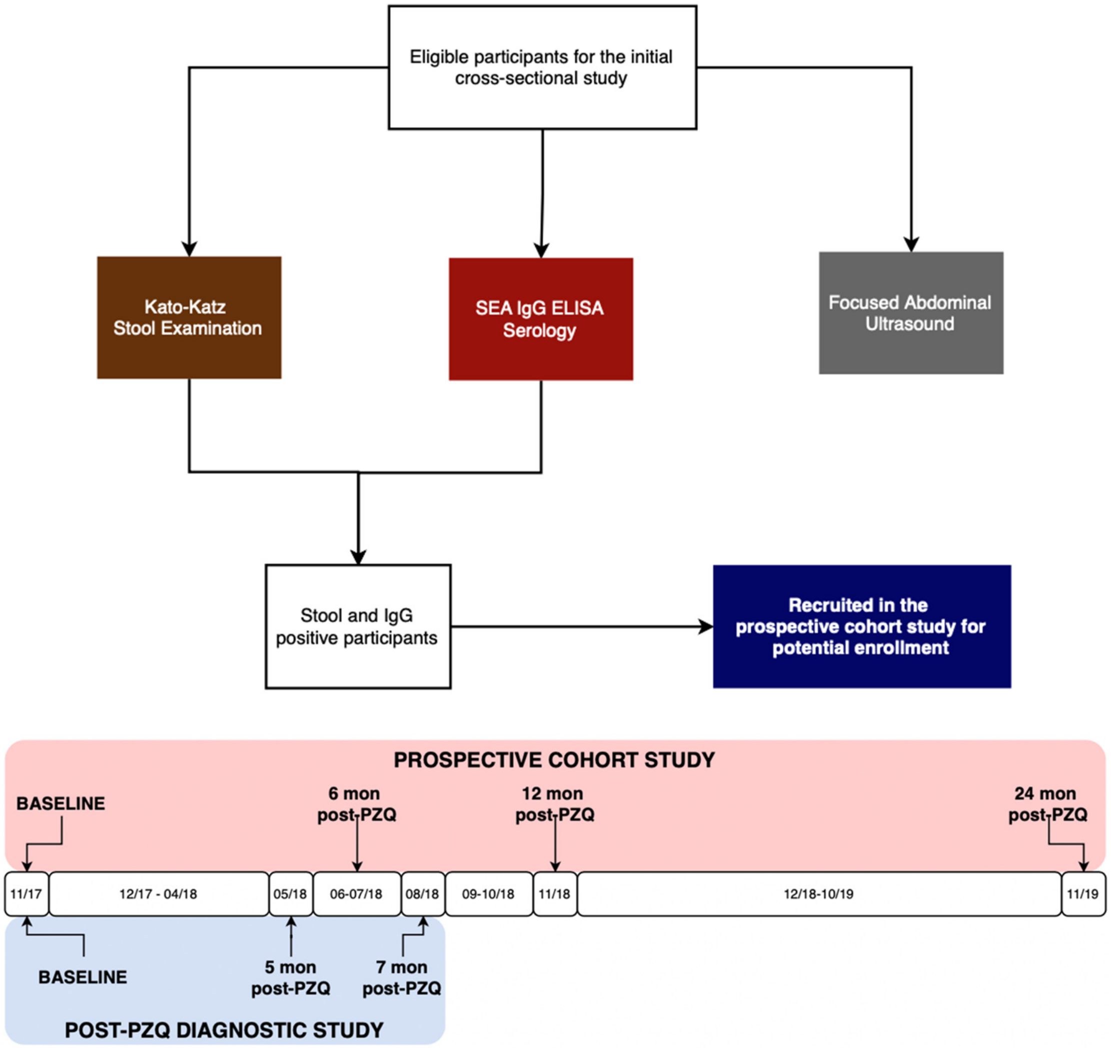

Supplementary Figure S1. Workflow for the prospective cohort study. **Abbreviations**: Praziquantel (PZQ), soluble egg antigen (SEA), Immunoglobulin G (IgG).

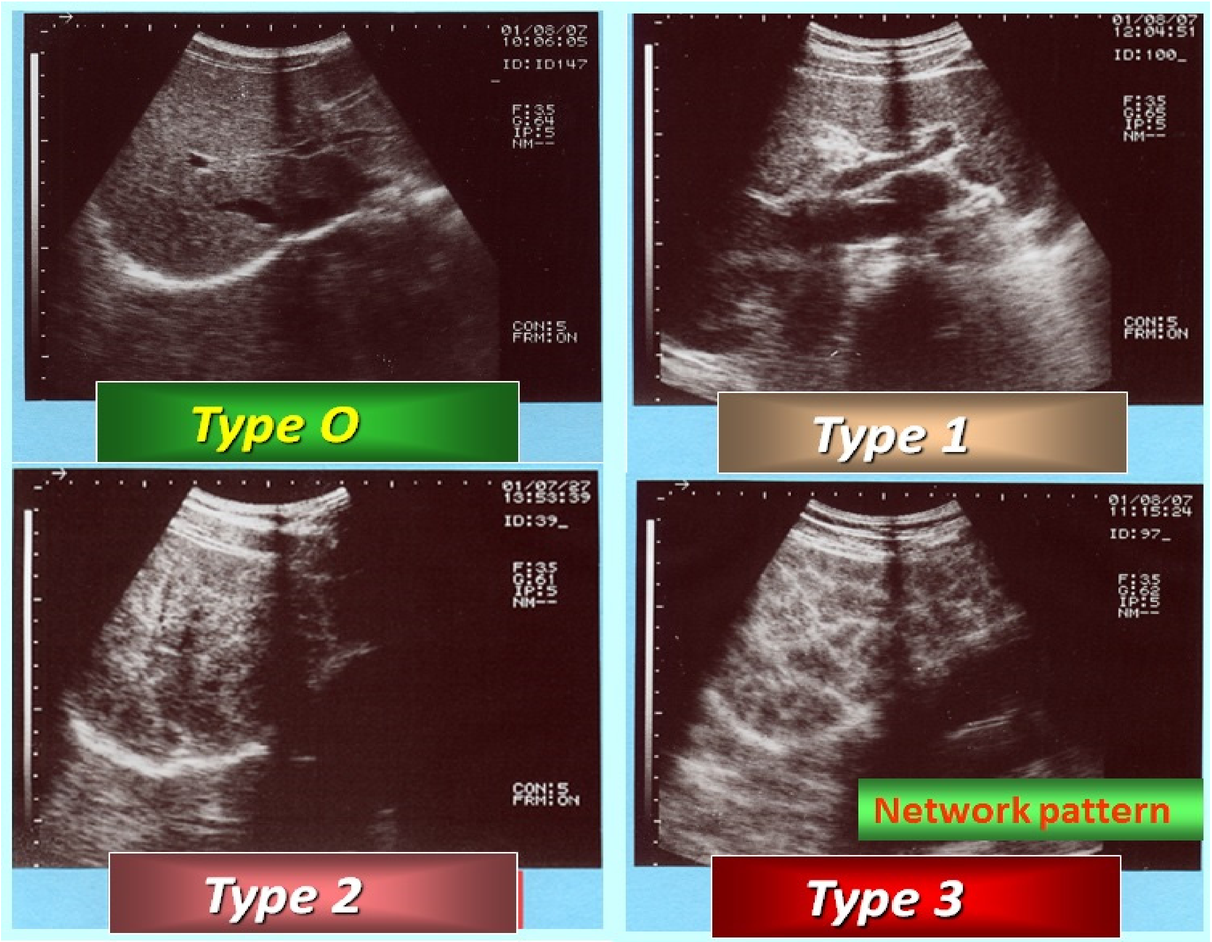

Supplementary Figure S2. Ohmae et al. (1992) US-based hepatic fibrosis severity. Images were obtained from Dr. Yuichi Chigusa and used with permission.

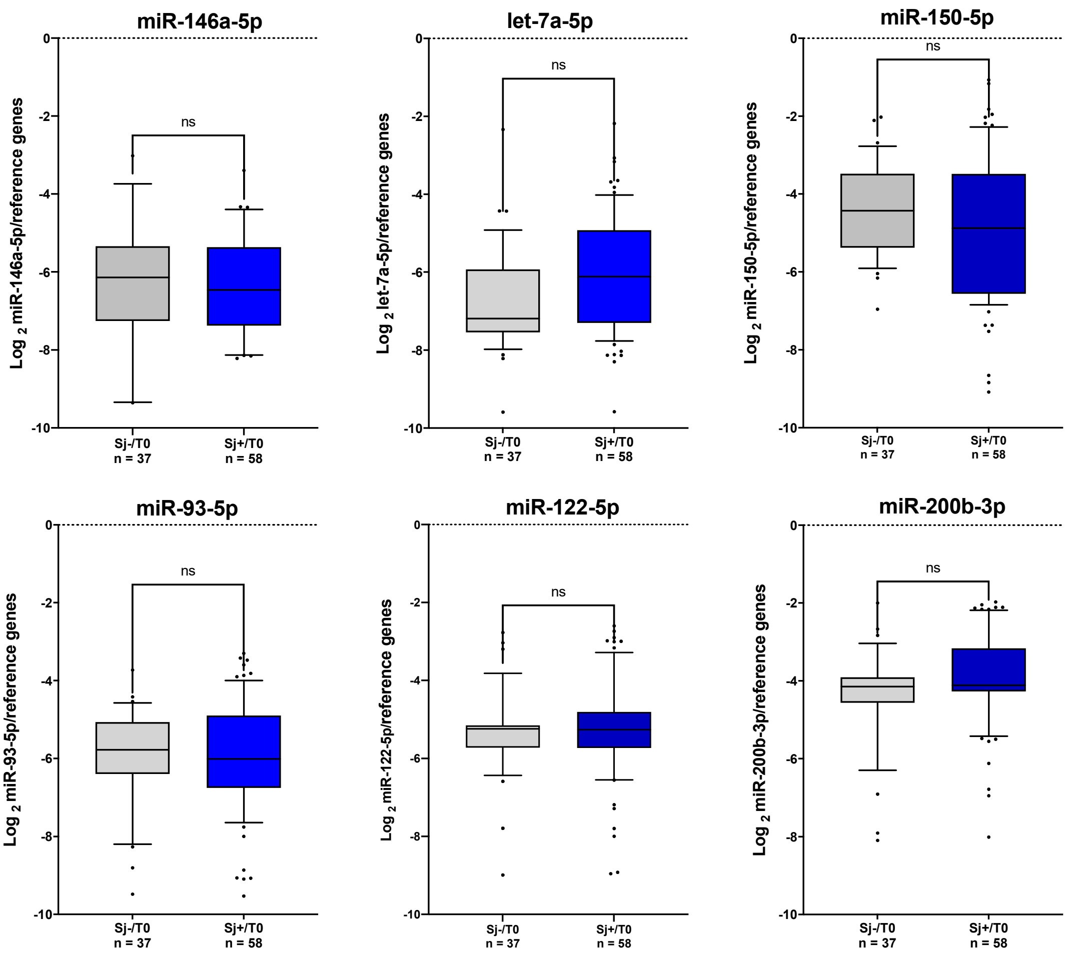

Supplementary Figure S3. Differentiation of participants with (Sj+/T0, *n* = 37) and without (Sj−/T0, *n* = 58) active schistosomiasis by serum levels of the six target miRNAs.

Note: Values were normalized using the average of the endogenous SNORD95 and the spiked-in UniSp6 reference miRNAs. The boxes represent the interquartile range, while the lines across the boxes indicate the median value. The hash marks above and below the boxes show the 90th and 10th percentiles for each group, respectively. Data were checked for normality and lognormality. Mann-Whitney test was used (ns – no significant difference).

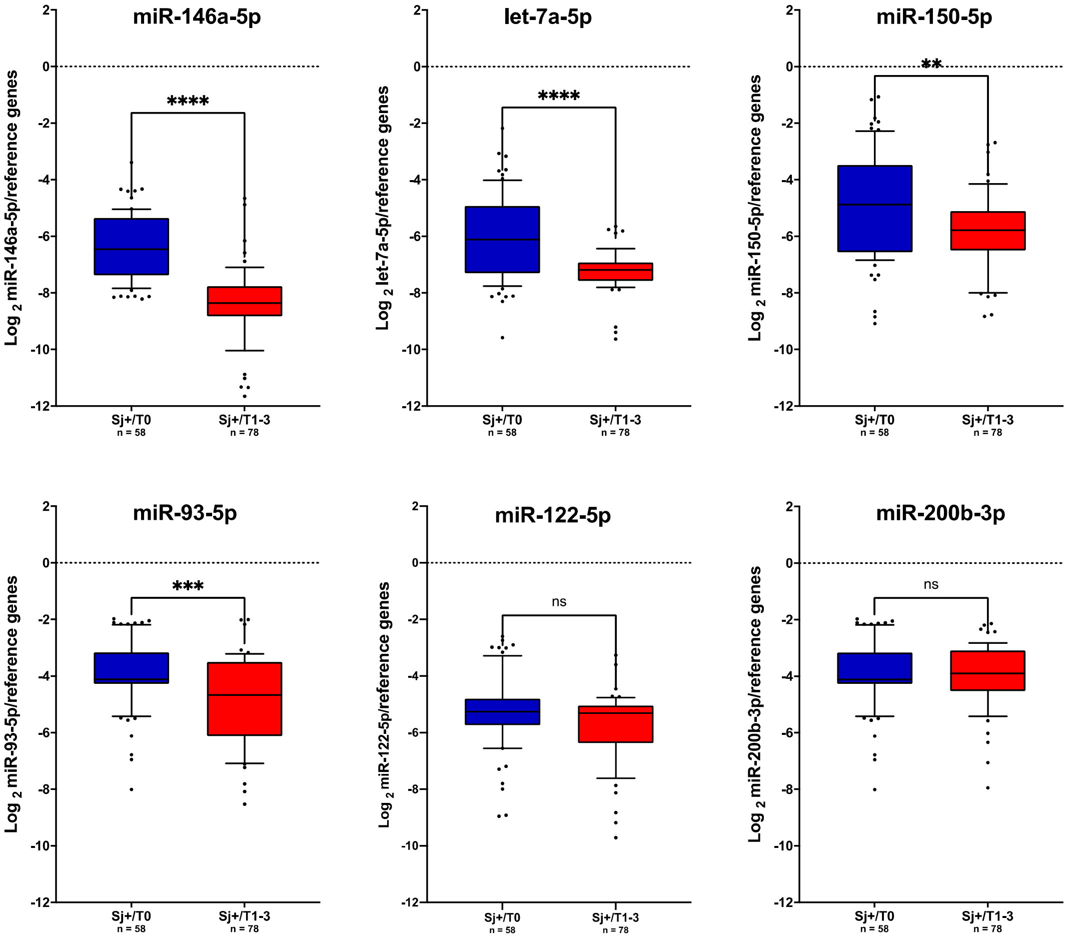

Supplementary Figure S4. Differentiation of chronic schistosomiasis patients with (Sj+/T1-3, *n* = 78) and without (Sj+/T0, *n* = 58) hepatic fibrosis by serum levels of the six target miRNAs.

Note: Values were normalized using the average of the endogenous SNORD95 and the spiked-in UniSp6 reference miRNAs. The boxes represent the interquartile range, while the lines across the boxes indicate the median value. The hash marks above and below the boxes show the 90th and 10th percentiles for each group, respectively. Data were checked for normality and lognormality. Mann-Whitney test was used (ns – no significant difference, ** $p < 0.01$, *** $p < 0.001$, and **** $p < 0.0001$).

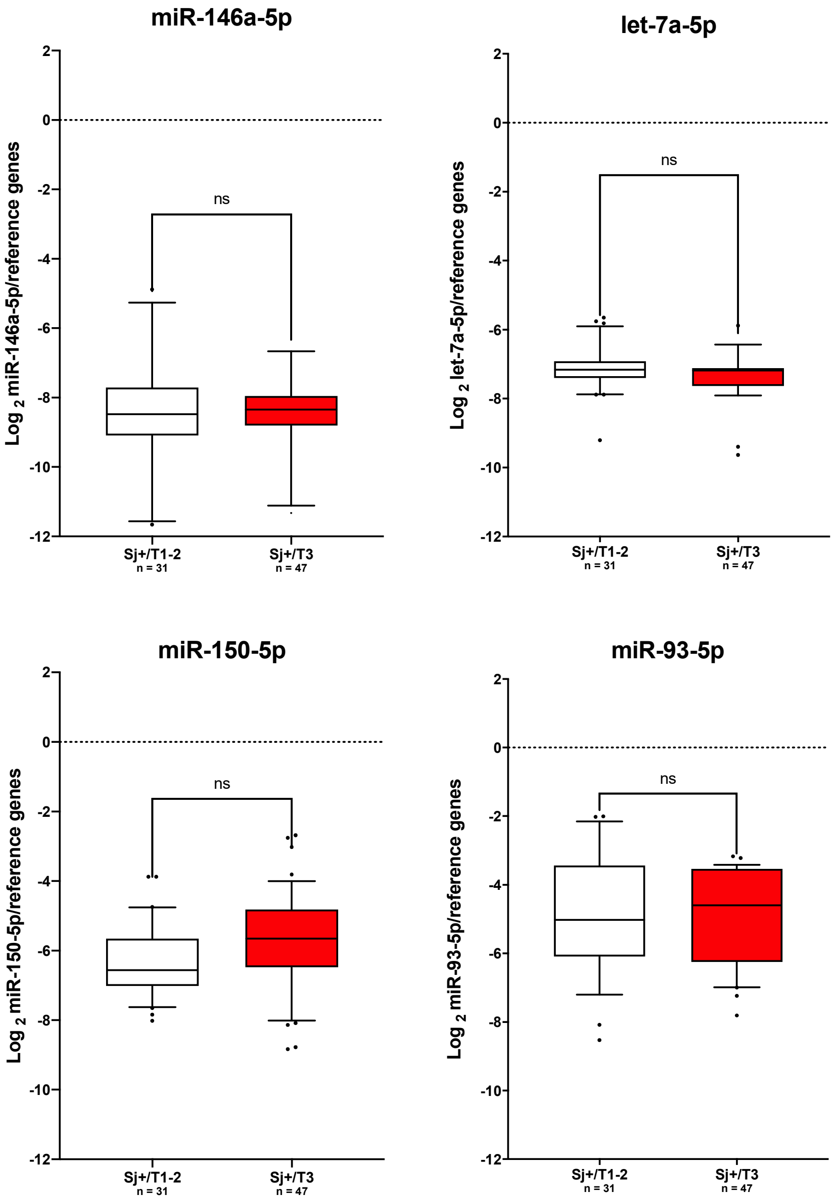

Supplementary Figure S5. Differentiation of chronic schistosomiasis patients with mild (Sj+/T1-2, *n* =31) and severe (Sj+/T3, *n* = 47) hepatic fibrosis by serum levels of the target miRNAs.

Note: Values were normalized using the average of the endogenous SNORD95 and the spiked-in UniSp6 reference miRNAs. The boxes represent the interquartile range, while the lines across the boxes indicate the median value. The hash marks above and below the boxes show the 90th and 10th percentiles for each group, respectively. Data were checked for normality and lognormality. Mann-Whitney test was used (ns – no significant difference).

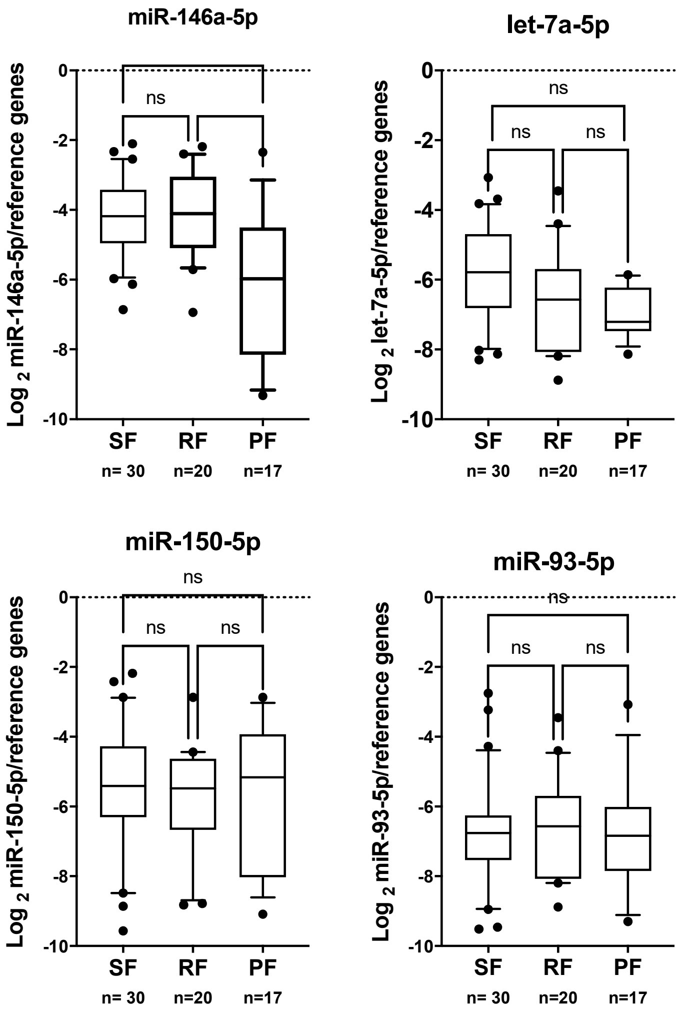

Supplementary Figure S6. Differentiation of patients with stable US findings (SF, *n* = 30), reversal of fibrosis (RF, *n* = 20), and progressive fibrosis (PF, *n* = 17) by baseline serum levels of the 6 target miRNAs.

Note: Values were normalized using the average of the endogenous SNORD95 and the spiked-in UniSp6 reference miRNAs. The boxes represent the interquartile range while the lines across the boxes indicate median value. The hash marks above and below the boxes show the 90th and 10th percentiles for each group, respectively. Data were checked for normality and lognormality. Ordinary one-way ANOVA with either Kruskal-Wallis (non-parametric) or Brown-Forsythe (parametric with unequal variance) tests was used (ns – no significant difference)
